# Efficacy and Safety of Switching from Adalimumab Originator to SB5 Biosimilar in Noninfectious Uveitis: Early Clinical Outcomes

**DOI:** 10.3390/jcm14228179

**Published:** 2025-11-18

**Authors:** Ji Hun Song, Chungwoon Kim, Yoo-Ri Chung, Hae Rang Kim

**Affiliations:** Department of Ophthalmology, Ajou University School of Medicine, Suwon 16499, Republic of Korea; uni427@naver.com (C.K.); khr1412@hanmail.net (H.R.K.)

**Keywords:** adalimumab, biosimilar, noninfectious uveitis, SB5, switching

## Abstract

**Background/Objectives:** To evaluate the real-world efficacy and safety of switching from adalimumab originator (Humira^®^, AbbVie) to SB5 biosimilar (Adalloce^®^, Samsung Bioepis) in patients with noninfectious uveitis. **Methods:** This retrospective study included 18 patients (32 eyes) who switched from adalimumab originator to SB5 for nonmedical reasons with at least 6 months follow-up. Clinical outcomes—best-corrected visual acuity (BCVA), intraocular pressure (IOP), anterior chamber (AC) cell grade, vitreous haze grade, central macular thickness (CMT), and macular volume (MV)—were assessed at baseline and 2, 4, and 6 months post-switch. Ultra-widefield fluorescein angiography (UWFA) findings were compared at approximately 4 months. Pre- versus post-switch comparisons employed Wilcoxon signed-rank tests. **Results:** Mean patient age was 45.7 ± 13.4 years, with mean follow-up of 16.8 ± 5.9 (range, 9–29) months. No significant changes were observed in BCVA, IOP, AC cell grade, vitreous haze grade, CMT, or MV at any timepoint versus baseline (all *p* > 0.05). No uveitis recurrence occurred based on predefined criteria including AC cell grade, vitreous haze grade, BCVA, and UWFA findings. Five patients (28%) re-switched to the originator after a mean of 24 (range, 4–64) weeks due to injection-site discomfort (n = 2) or extremity rashes (n = 3). No other adverse events were observed. **Conclusions:** Switching from adalimumab originator to SB5 biosimilar maintained clinical stability with comparable efficacy and safety in patients with noninfectious uveitis, supporting its use as a cost-effective alternative.

## 1. Introduction

Noninfectious uveitis is a chronic inflammatory condition requiring long-term immunosuppression to prevent vision-threatening complications. Biologic agents, particularly tumor necrosis factor-α (TNF-α) inhibitors, have revolutionized uveitis management by targeting specific inflammatory pathways [1]. Adalimumab, a fully humanized IgG1 monoclonal antibody with high affinity for TNF-α, has demonstrated robust efficacy and safety in treating noninfectious uveitis and is widely used in clinical practice [2,3,4,5,6,7,8,9,10,11].

Despite their therapeutic benefits, the high cost of biologic agents poses a significant barrier to accessibility and long-term sustainability for patients and healthcare providers [12]. Biosimilars—highly similar versions of approved biologic agents—have emerged as cost-effective alternatives following the expiration of originator patents [13,14]. In Korea, two adalimumab biosimilars, SB5 (Adalloce^®^, Samsung Bioepis, Incheon, Republic of Korea) and Yuflyma^®^ (Celltrion, Incheon, Republic of Korea), received regulatory approval in 2017 and 2021, respectively, for treating noninfectious uveitis (excluding anterior uveitis) in adult patients. However, theoretical concerns regarding potential loss of efficacy, altered immunogenicity, or safety differences following biosimilar switching have been raised [15,16].

Most previous real-world studies on biosimilar switching have focused on systemic rheumatological conditions, including inflammatory bowel disease, ankylosing spondylitis, psoriatic arthritis, and rheumatoid arthritis [17]. Studies specifically evaluating adalimumab biosimilar switching in noninfectious uveitis are limited. Sota et al. [18] demonstrated the effectiveness of Imraldi^®^ (Biogen, Berkshire, UK) in patients with noninfectious uveitis. Murray et al. [19] reported the safety and efficacy of switching from adalimumab originator (Humira^®^, AbbVie Inc., North Chicago, IL, USA) to ABP 501 biosimilar (Amgevita^®^, Amgen Europe B.V., Breda, The Netherlands) in uveitis management. Song et al. [20] revealed the efficacy and safety of switching from Humira^®^ to SB5 (Adalloce^®^) in a small cohort of patients with noninfectious uveitis with limited follow-up duration.

This study aimed to further evaluate the real-world efficacy and safety of switching from adalimumab originator (Humira^®^) to SB5 biosimilar (Adalloce^®^) in patients with noninfectious uveitis.

## 2. Materials and Methods

The medical records of 18 patients (32 eyes) with noninfectious uveitis who were switched from an adalimumab originator to SB5 for nonmedical reasons were retrospectively reviewed and analyzed. Only patients with at least 6 months of follow-up at Ajou University Hospital between April 2023 and August 2025 were included. Patients who initiated biologics therapy with SB5 (i.e., biosimilar-naïve patients) were excluded. This study complied with the tenets of the Declaration of Helsinki and was approved by the Institutional Review Board (IRB) of Ajou University Hospital (approval no. AJOUIRB-DB-2025-207). SB5 (Adalloce^®^) was introduced at our institution in February 2023, and the first patient fulfilling the inclusion criteria was switched to SB5 in April 2023. The clinical observation period (switching and follow-up) for eligible cases spanned between April 2023 and August 2025. IRB approval was obtained in April 2025, prior to accessing and analyzing any retrospective medical records. Following IRB approval, all retrospective data extraction and statistical analysis were performed.

Detailed inclusion and exclusion criteria were as follows. Inclusion criteria were: (1) diagnosis of noninfectious posterior or panuveitis; (2) previous treatment with adalimumab originator (Humira^®^); (3) switching to SB5 biosimilar (Adalloce^®^) for nonmedical reasons; (4) minimum follow-up of 6 months after switching; and (5) availability of comprehensive ophthalmic examination records. Exclusion criteria included: (1) patients who initiated biologic therapy with SB5 (biosimilar-naïve patients); (2) infectious uveitis or isolated anterior uveitis; (3) incomplete medical records or follow-up less than 6 months; and (4) concurrent ocular diseases that could significantly affect visual outcomes or confound clinical outcome analysis.

The following demographic, clinical, and therapeutic data were collected for analysis: sex, age, laterality, anatomical pattern of uveitis, associated systemic disease, and concomitant and previous immunomodulatory treatments. Prior to initiating adalimumab therapy, all patients underwent assessment of systemic disease activity related to the underlying etiology of uveitis. Systemic manifestations of Behçet’s disease (e.g., oral/genital ulcers, skin lesions, arthritis), neurologic/auditory/integumentary involvement in Vogt–Koyanagi–Harada disease, and possible connective tissue or autoimmune disease in idiopathic uveitis or posterior scleritis were evaluated. Treatment decisions were based on ocular inflammation status rather than systemic disease manifestations. To evaluate the clinical efficacy and safety of the biosimilar switch, primary outcome measures included best-corrected visual acuity (BCVA, logarithm of the minimum angle of resolution [logMAR]), intraocular pressure (IOP, mmHg), anterior chamber (AC) cell grade, vitreous haze grade, central macular thickness (CMT, μm), and macular volume (MV, mm^3^) at baseline (pre-switching) and at 2, 4, and 6 months post-switching. BCVA was measured using a Snellen chart and converted to logMAR values for analysis. For patients with severely reduced vision—such as “counting fingers,” “hand motion,” or “light perception”—corresponding numerical values were assigned for statistical analysis. AC cell grade was assessed according to the Standardization of Uveitis Nomenclature criteria [21]. Vitreous haze grade was evaluated according to Nussenblatt’s method [22,23]. CMT and MV were measured using spectral-domain optical coherence tomography (Heidelberg Spectralis, Heidelberg Engineering, Heidelberg, Germany). CMT was defined as the average retinal thickness within a 1.0 mm diameter centered on the fovea. Furthermore, ultra-widefield fluorescein angiography (UWFA) findings were compared at approximately 4 months post-switching. All UWFA images were independently interpreted by two investigators (J.H.S. and C.K.). In cases of any discrepancy, consensus was reached through discussion.

Adalimumab was administered at a dose of 40 mg subcutaneously at 2-week intervals, which was consistent with the dosing regimen both before and after switching. Uveitis recurrence was defined as: (1) an increase of two or more steps in AC cell grade [21]; (2) an increase of two or more steps in vitreous haze grade [22,23]; (3) a decrease of three or more lines (0.3 logMAR units) in BCVA [24]; or (4) definite aggravation of vascular leakage in zone I or II on UWFA.

To explore whether concomitant systemic immunomodulatory therapy influenced treatment outcomes, patients were categorized into four subgroups based on their regimen at the time of switching: (1) methotrexate monotherapy, (2) mycophenolate mofetil with or without low-dose prednisone, (3) combination therapy with mycophenolate mofetil and cyclosporine with or without low-dose prednisone, and (4) low-dose prednisone alone. Clinical outcomes were compared between baseline and 6 months post-switch using the Wilcoxon signed-rank test.

Prior to initiating adalimumab therapy, all patients underwent comprehensive safety evaluation. Laboratory tests were performed to exclude infectious uveitis, including serologic testing for herpes simplex virus, varicella zoster virus, cytomegalovirus, human immunodeficiency virus, toxoplasmosis, toxocariasis, and syphilis. Screening for latent tuberculosis was conducted using interferon-gamma release assay (IGRA) and chest X-ray. In IGRA-positive patients, prophylactic anti-tubercular therapy was completed prior to starting adalimumab after consultation with an infectious disease specialist. Hepatitis B and C serologies were also obtained; patients with positive hepatitis B or C serology were co-managed with hepatology and initiated on antiviral therapy if indicated. Medical history was reviewed for malignancy, demyelinating disease, and congestive heart failure. Screening for autoimmune disease and inflammatory status included antinuclear antibody, rheumatoid factor, erythrocyte sedimentation rate, and C-reactive protein. Baseline laboratory testing included complete blood count (CBC) and comprehensive metabolic panel with liver and kidney function tests to document hematologic, hepatic, and renal status before initiating biologic therapy.

Safety monitoring consisted of structured review of systems at every visit and routine laboratory testing (complete blood count and chemistry panel, including liver function tests) every 16 weeks to evaluate hematologic or hepatic adverse effects. Infectious complications and injection-site reactions were assessed at each visit, and any patient-reported adverse events were recorded.

Statistical analyses were performed using IBM SPSS Statistics for Windows, version 27 (IBM Corp., Armonk, NY, USA). Pre- and post-switching data were compared using the Wilcoxon signed-rank test. A *p*-value < 0.05 was considered statistically significant.

## 3. Results

A total of 18 patients (32 eyes) with noninfectious uveitis who switched from an adalimumab originator to SB5 for nonmedical reasons were included in this study. The cohort comprised 8 females and 10 males with a mean age of 45.7 ± 13.4 years. The mean follow-up duration was 16.8 ± 5.9 (range, 9–29) months. Uveitis was unilateral in 4 patients and bilateral in 14 patients. Regarding anatomical classification, 2 patients had posterior uveitis and 16 had panuveitis.

In terms of etiology, 7 patients had idiopathic uveitis, 7 had Behçet’s disease, 3 had Vogt–Koyanagi–Harada disease, and 1 was diagnosed with posterior scleritis. Interferon gamma release assay (IGRA) testing revealed T-SPOT positivity in 5 patients; among these, 2 had Behçet’s disease and 3 had idiopathic uveitis unrelated to tuberculosis. All patients with IGRA positivity received anti-tubercular therapy as a preventive measure before initiating adalimumab treatment. Regarding systemic disease control, all patients with systemic diseases (Behçet’s disease or Vogt–Koyanagi–Harada disease, n = 10, 56%) had stable systemic disease activity at both the initiation of adalimumab and the time of switching to SB5. Treatment decisions were based solely on ocular inflammatory status, and the switch to SB5 was performed exclusively for nonmedical reasons while systemic disease activity remained controlled.

Seventeen patients continued oral immunomodulatory medications after switching to SB5, whereas 1 patient did not receive concomitant immunomodulatory therapy either before or after switching. Oral immunomodulatory medications included mycophenolate mofetil, cyclosporine, or methotrexate, with or without low-dose oral corticosteroids (5–7.5 mg daily). The demographic and clinical characteristics of all patients are summarized in Table 1.

**Figure 1:**
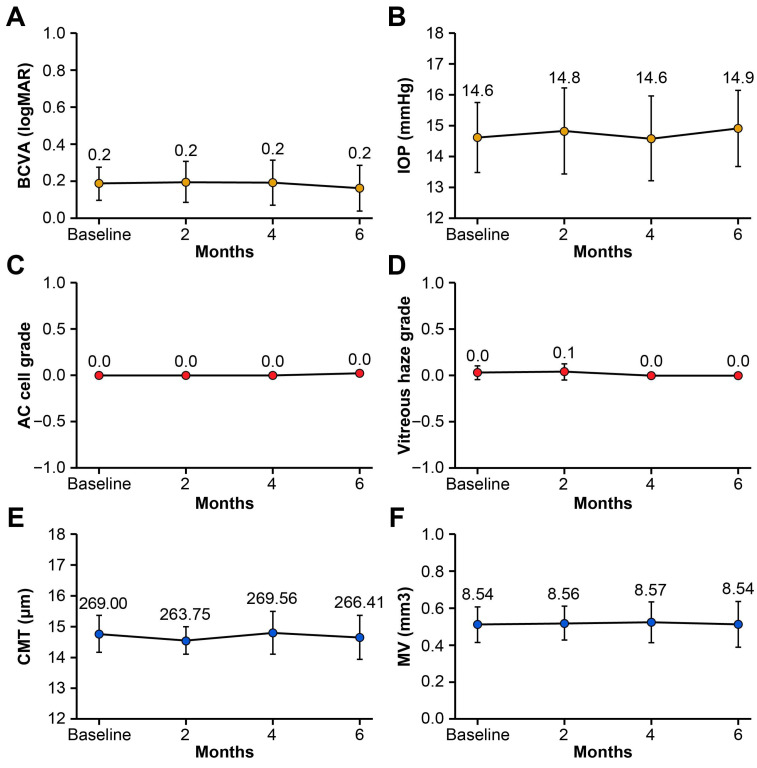
Changes in (**A**) best-corrected visual acuity (BCVA, logMAR), (**B**) intraocular pressure (IOP, mmHg), (**C**) anterior chamber (AC) cell grade, (**D**) vitreous haze grade, (**E**) central macular thickness (CMT, μm), and (**F**) macular volume (MV, mm^3^) at baseline and at 2, 4, and 6 months after switching from the adalimumab originator to SB5. Points indicate means, and error bars indicate 95% confidence intervals.

**Figure 2:**
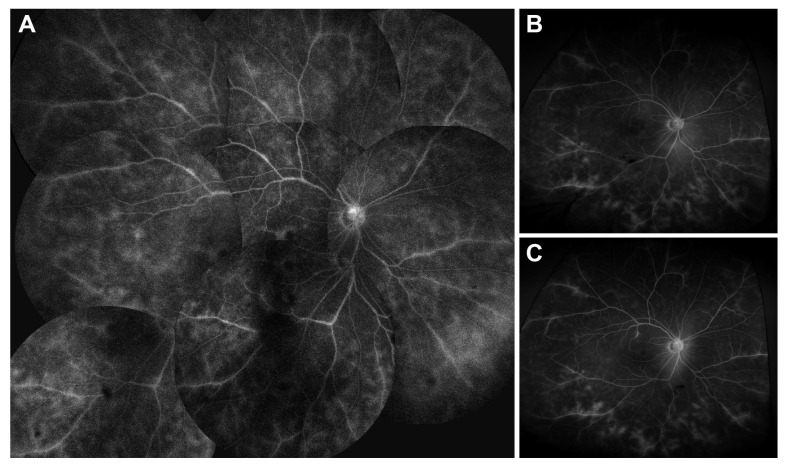
Fluorescein angiography (FA) of a patient with Behçet’s disease-associated panuveitis. (**A**) Peak phase of angiogram before treatment initiation with the adalimumab originator (Humira^®^) demonstrates severe diffuse fern-like vascular leakage and cystoid macular edema. (**B**) After treatment with adalimumab originator, marked resolution of vascular leakage and improvement in macular edema are evident. (**C**) Four months following the switch to the adalimumab biosimilar (SB5, Adalloce^®^), FA findings remain stable without worsening, indicating maintained disease control.

No significant changes in BCVA (logMAR), IOP (mmHg), AC cell grade, vitreous haze grade, CMT (μm), or MV (mm^3^) were observed at 2, 4, or 6 months following the switch compared with baseline (all *p* > 0.05, Wilcoxon signed-rank test; Table 2 and Figure 1). No uveitis recurrences occurred based on the predefined criteria (AC cell grade, vitreous haze grade, and BCVA). In our cohort, because all patients had stable disease activity at the time of switching to SB5, there were no detectable changes in UWFA findings before and after switching, and no inter-grader disagreement occurred. Subgroup analysis based on systemic immunomodulatory regimen (methotrexate monotherapy, mycophenolate mofetil ± low-dose prednisone, combination therapy with mycophenolate mofetil and cyclosporine ± low-dose prednisone, or low-dose prednisone only) demonstrated no statistically significant changes in BCVA, IOP, AC cell grade, vitreous haze grade, CMT, or macular volume between baseline and 6 months post-switch (all *p* > 0.05, Wilcoxon signed-rank test). At 6 months post-switch, four patients (22%) were able to taper or discontinue systemic glucocorticoids, and one patient (6%) discontinued concomitant methotrexate therapy. This tapering occurred as part of routine disease management, as intraocular inflammation remained stably controlled throughout the follow-up period. A representative case of Behçet’s disease-associated panuveitis demonstrating maintained disease control after switching from an adalimumab originator to SB5 is depicted in Figure 2.

Routine laboratory monitoring (CBC and chemistry panel, including liver function tests every 16 weeks) revealed no hematologic or hepatic abnormalities during SB5 treatment. No patient developed opportunistic infection, tuberculosis reactivation, or reactivation of hepatitis B or C. Adverse events consisted of mild injection-site discomfort (n = 2) and transient extremity rashes (n = 3). All events were documented during scheduled clinic visits based on clinical evaluation and laboratory results, rather than patient self-report alone.

**Figure 3:**
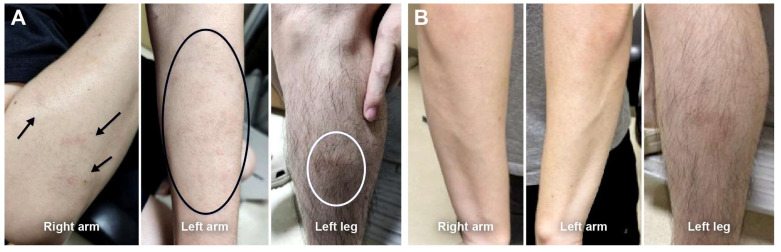
Representative case of an upper- and lower extremity rash after switching from the adalimumab originator to SB5. (**A**) At 9 weeks post-switching, eczematous eruptions are noted to develop on the bilateral arms and legs (arrows and circles). (**B**) At 4 weeks after switching back to the adalimumab originator, cutaneous lesions demonstrate marked improvement.

Five patients (28%) re-switched to the adalimumab originator (Humira^®^) after a mean duration of 24 (range, 4–64) weeks. Reasons for re-switching included injection-site discomfort (n = 2) and extremity rashes (n = 3); among the latter, two patients experienced rashes on both upper and lower extremities, and one patient had rashes confined to the lower extremities. These rashes were identified during scheduled clinic visits through structured review of systems and clinical examination. The rashes were characterized by erythematous papular eruptions without mucosal involvement, distinct from injection-site reactions. SB5 was discontinued immediately upon rash identification, and all rashes resolved completely without requiring antibiotics or systemic immunosuppression. Resolution of the rash after re-switching to the originator suggests drug-specific hypersensitivity to the SB5 biosimilar rather than an infectious etiology. No other systemic or ocular adverse events were observed following the switch to SB5 (Table 3). A representative case of an extremity rash is illustrated in Figure 3.

## 4. Discussion

Despite the increasing clinical use of biosimilars, real-world evidence on their efficacy and safety in noninfectious uveitis remains limited. To address this knowledge gap, we evaluated the clinical efficacy and safety of switching from the adalimumab originator (Humira^®^) to the SB5 biosimilar (Adalloce^®^) in patients with noninfectious uveitis.

Several studies have evaluated adalimumab biosimilars in treatment-naïve patients. A retrospective study by Sota et al. [18] involving 26 adalimumab-naïve adults with noninfectious uveitis evaluated the efficacy of the adalimumab biosimilar Imraldi^®^. Treatment with Imraldi^®^ was associated with significantly decreased uveitis relapse and retinal vasculitis rates, as well as improved visual acuity. Moreover, a marked glucocorticoid-sparing effect and a high drug-retention rate were observed. No new ocular complications were reported during the treatment period. Similarly, a cohort study in Iran by Soheilian et al. [25] involving 48 anti-TNF-α-naïve patients with Behçet’s uveitis demonstrated the clinical effectiveness of the adalimumab biosimilar CinnoRA^®^ (CinnaGen Company, Alborz, Iran). The study revealed significant improvements in visual acuity and AC cell grade, as well as significantly decreased vitreous haze grade.

Studies evaluating the switch from originator to biosimilar have also demonstrated comparable outcomes. A retrospective study by Song et al. [20] involving 15 patients with noninfectious uveitis followed for a mean of 10 months reported no significant differences in clinical outcomes after switching from the adalimumab originator (Humira^®^) to the SB5 biosimilar (Adalloce^®^), with no uveitis recurrences observed. By comparison, our study provides longer-term safety and efficacy data with a mean follow-up of 16.8 months (range, 9–29 months), which is particularly important for chronic conditions like noninfectious uveitis that require sustained disease control. Four patients (27%) re-switched to the originator due to injection-related discomfort (n = 3) and technical difficulty with the new device (n = 1), with no other serious adverse events reported. Similarly, a multicenter retrospective study by Murray et al. [19] involving 102 patients with uveitis showed no significant change in uveitis flare rates following the switch from an adalimumab originator (Humira^®^) to ABP 501 biosimilar (Amgevita^®^); additionally, the rate of elevated IOP decreased after the switch. Following the switch to ABP 501, 20 patients reported adverse events, with injection-site pain being the most frequent (13/20, 65%); notably, 92.3% (12/13) of these cases occurred in pediatric patients. Other reported adverse events included localized cutaneous reactions, patient-reported visual difficulties, and injector-related technical issues [19]. Of the 102 patients, 24 (24%) re-switched to Humira^®^ (15 pediatric, 9 adult patients), predominantly at the patient’s request due to injection pain or injector difficulties; one patient re-switched due to an allergic reaction to the biosimilar [19]. Additionally, Fabiani et al. [26] assessed uveitis flare rates in 37 patients with noninfectious uveitis who switched from various anti-TNF-α originators to their respective biosimilars, including Imraldi^®^ (adalimumab biosimilar), Flixabi^®^ (SB2, infliximab biosimilar, Samsung Bioepis, Incheon, Republic of Korea), Inflectra^®^ (CT-P13, infliximab biosimilar, Pfizer Inc., New York, NY, USA), and Benepali^®^ (SB4, etanercept biosimilar, Samsung Bioepis, Incheon, Republic of Korea). Flare rates showed no significant differences at more than 12 months post-switching. Moreover, the risk of post-switch uveitis flares appeared independent of baseline disease control. No serious adverse events were reported following the switch.

In our study, no significant changes were observed in BCVA, IOP, AC cell grade, vitreous haze grade, CMT, or MV at 2, 4, and 6 months after the switch compared with baseline (all *p* > 0.05, Wilcoxon signed-rank test). To comprehensively assess disease activity, we employed both traditional clinical parameters and objective imaging with UWFA, which provides superior visualization of peripheral retinal vasculature compared with conventional fluorescein angiography. This multimodal approach enhances the reliability of our findings and ensures that subtle disease activity was not overlooked. No uveitis recurrences occurred during follow-up based on these comprehensive predefined criteria (AC cell grade, vitreous haze grade, BCVA, and UWFA findings). These findings are consistent with previous studies [18,19,20,25,26] demonstrating the effectiveness of adalimumab biosimilars in treating noninfectious uveitis. It is important to note that the glucocorticoid-sparing effect observed in four patients was not attributable to superior anti-inflammatory efficacy of SB5 compared to the originator. Rather, because the switch occurred while inflammation was already well controlled on the originator and disease activity remained stable throughout the follow-up period, successful maintenance of disease control allowed for routine gradual dose reduction. Additionally, our study contributes real-world data from an Asian population, adding geographic diversity to the predominantly Western literature on biosimilar switching in uveitis. This is particularly relevant given potential ethnic differences in drug metabolism, immune response, and disease phenotypes that may affect treatment outcomes.

In our cohort, 5 patients (28%) re-switched to the adalimumab originator: 2 patients due to injection-site discomfort and 3 due to extremity rashes (2 involving both upper and lower extremities and 1 confined to the lower extremities). The reasons for re-switching were comparable to those reported in previous studies [19,20] and were not associated with uveitis recurrence. Resolution of the rash after re-switching to the originator suggests drug-specific hypersensitivity to the SB5 adalimumab biosimilar. No other systemic or ocular adverse events developed following the switch to SB5, consistent with previous studies [18,20]. These findings provide reassurance for clinicians and patients regarding the safety of switching to SB5.

Previous real-world studies have reported injection-site pain or injector/device difficulties as major reasons for re-switching to the adalimumab originator following biosimilar switch. Song et al. [20] reported that 27% (4/15) of patients re-switched after initiating SB5, with 20% (3/15) re-switching due to injection-related discomfort. Murray et al. [19] revealed that 24% (24/102) of patients re-switched to Humira^®^; of these, 23% (23/102) did so at the patient’s request due to injection pain or injector difficulties, and 1 patient re-switched due to an allergic reaction. By contrast, in our cohort, only 11% (2/18) of patients re-switched due to injection-site discomfort, a proportion lower than that in previous studies; instead, 17% (3/18) re-switched due to extremity rashes.

It is noteworthy that on June 1, 2023, a high-concentration, citrate-free formulation of SB5 (40 mg/0.4 mL) was introduced, replacing the prior low-concentration formulation (40 mg/0.8 mL). In our cohort, the proportion of injection-site discomfort-related re-switching (2/18, 11%) was lower than that reported in previous studies (e.g., 3/15 [20%] in Song et al. [20]; 23/102 [23%] in Murray et al. [19]). This reduction may be attributable to improved local tolerability of the citrate-free, high-concentration formulation. Supporting this interpretation, a randomized pharmacokinetic study in patients with rheumatoid arthritis demonstrated that the high-concentration formulation resulted in significantly less injection-site pain and discomfort compared with the standard low-concentration formulation [27]. However, given the retrospective nature of our study, a causal relationship cannot be definitively established, and other potential contributing factors—such as differences in injection devices or reporting biases—cannot be excluded.

This study has several limitations that warrant consideration. First, the retrospective design inherently limits causal inference and may introduce selection bias. The retrospective nature of the study also meant that standardized and protocol-driven assessment for potential adverse effects was not feasible, and safety evaluations were performed only at routine clinic visits. Second, the relatively small sample size (18 patients, 32 eyes) may limit the statistical power to detect subtle differences in clinical outcomes and rare adverse events. Additionally, the follow-up period, while adequate for detecting early treatment responses and acute adverse events, may not capture long-term outcomes such as late-onset complications, delayed hypersensitivity reactions, or changes in drug efficacy over extended periods. Third, the absence of a concurrent control group (patients continuing on the originator) precludes direct comparative analysis of treatment outcomes. Fourth, as a single-center study conducted at a tertiary referral center, the findings may not be fully generalizable to other healthcare settings or patient populations with different demographic characteristics. The heterogeneous etiology of noninfectious uveitis in our cohort (idiopathic, Behçet’s disease, Vogt–Koyanagi–Harada disease, and posterior scleritis) may introduce variability in treatment response, although this heterogeneity also reflects real-world clinical practice. Furthermore, the concomitant use of oral immunomodulatory medications in most patients (17/18) makes it difficult to isolate the specific contribution of SB5 to the observed outcomes. Another limitation is that all patients had quiescent inflammation at the time of switching (anterior chamber cell grade 0 and vitreous haze grade 0), which may have reduced the likelihood of detecting subtle differences in treatment efficacy between the originator and the biosimilar. Finally, the nonmedical reasons for switching (administrative or economic factors) may differ from medically indicated switches, potentially affecting the applicability of our findings to other switching scenarios.

Despite these limitations, our study demonstrates that switching from adalimumab originator to SB5 biosimilar maintains clinical stability with comparable efficacy and safety profiles in patients with noninfectious uveitis. Importantly, our findings suggest potentially improved local tolerability with the newer citrate-free, high-concentration formulation of SB5, as evidenced by a lower rate of injection-site discomfort-related re-switching compared with previous studies. These results support the use of SB5 as a safe and cost-effective therapeutic alternative in the real-world management of noninfectious uveitis.

## 5. Conclusions

This retrospective real-world study demonstrates that switching from adalimumab originator (Humira^®^) to SB5 biosimilar (Adalloce^®^) maintains clinical stability with comparable efficacy and safety in patients with noninfectious uveitis over a mean follow-up of 16.8 months. Notably, the lower rate of re-switching due to injection-site discomfort in our cohort compared with previous studies potentially reflects improved local tolerability of the citrate-free, high-concentration formulation introduced in 2023. These findings provide reassurance for clinicians and patients regarding biosimilar switching and support the use of SB5 as a cost-effective therapeutic alternative in the management of noninfectious uveitis. Further studies with a larger number of patients with various uveitis conditions and longer follow-up periods are warranted to confirm these findings.

## Figures and Tables

**Table 1:** Demographics and clinical characteristics of the enrolled 18 patients.

Variables	Outcome
**Sex** (F:M)	8:10
**Age** (years)	45.7 ± 13.4
**Follow-up duration** (months)	16.8 ± 5.9
**Laterality** (Unilateral:Bilateral)	4:14
**Anatomical pattern of uveitis**	
Posterior uveitis	2
Panuveitis	16
**Diagnosis**	
Idiopathic	7
Behçet’s disease	7
Vogt–Koyanagi–Harada disease	3
Posterior scleritis	1
**Positive IGRA**	5
**Concomitant oral IMT** (Continue:Stop)	17:0

**Abbreviation:** IGRA, interferon gamma release assay; IMT, immunomodulatory medication.

**Table 2:** Clinical outcomes at baseline and at 2, 4, and 6 months post-SB5 switching.

	Pre-Switch	Post-2 Months	Post-4 Months	Post-6 Months
BCVA (logMAR)	0.2 ± 0.2	0.2 ± 0.3	0.2 ± 0.3	0.2 ± 0.3
IOP (mmHg)	14.6 ± 3.1	14.8 ± 3.6	14.6 ± 3.5	14.9 ± 3.2
AC cell grade	0.0 ± 0.0	0.0 ± 0.0	0.0 ± 0.0	0.0 ± 0.1
Vitreous haze grade	0.0 ± 0.2	0.1 ± 0.2	0.0 ± 0.1	0.0 ± 0.1
CMT (μm)	269.00 ± 41.10	263.75 ± 28.90	269.56 ± 43.78	266.41 ± 44.49
MV (mm^3^)	8.54 ± 0.81	8.56 ± 0.74	8.57 ± 0.84	8.54 ± 0.95

**Abbreviations:** BCVA, best-corrected visual acuity; IOP, intraocular pressure; AC, anterior chamber; CMT, central macular thickness; MV, macular volume; logMAR, logarithm of the minimum angle of resolution.

**Table 3:** Patients who switched back to the adalimumab originator (Humira^®^), including reasons and timing of re-switching.

Patient No.	Duration on SB5 Treatment (Weeks)	Reason	Outcome After back Switching
P01	8	Injection site discomfort	Resolved
P02	4	Injection site discomfort	Resolved
P03	8	Lower extremity rash	Resolved
P04	36	Upper and lower extremity rash	Resolved
P05	64	Upper and lower extremity rash	Resolved

No other adverse events are reported in the remaining 13 patients (72%).

## Data Availability

The data presented in this study are available from the corresponding author upon reasonable request.

## Abbreviations

The following abbreviations are used in this manuscript:

AC	Anterior Chamber
BCVA	Best-Corrected Visual Acuity
CBC	Complete Blood Count
CMT	Central Macular Thickness
IGRA	Interferon Gamma Release Assay
IOP	Intraocular Pressure
logMAR	logarithm of the minimum angle of resolution
MV	Macular Volume
OCT	Optical Coherence Tomography
SUN	Standardization of Uveitis Nomenclature
UWFA	Ultra-Widefield Fluorescein Angiography
